# Supplementary material for: Unravelling the therapeutic potential of dual TGFβ-1 and CXCR4 inhibition in breast cancer using computational strategies
Source: PLoS One. 2025 Dec 5;20(12):e0323665. doi: 10.1371/journal.pone.0323665 (PMC12680324; doi:10.1371/journal.pone.0323665)
Supplement: Attachment — Submitted filename: Review comments for PLOS one.docx [file pone.0323665.s001.docx]

Upon reviewing the manuscript entitled "Exploring Soluble Mediators in Breast Cancer: Expression Dynamics of Transforming Growth Factor Beta 1 and C-X-C Chemokine Receptor Type 4 and the Therapeutic Opportunities," I have pinpointed a number of minor improvements that the authors could make-in order to enhance the general quality of the paper. The suggested revisions focus on improving the overall clarity, data interpretation, and presentation of the manuscript, ultimately enhancing its readability and scientific rigor.

1. Why were D4476 and AMD3100 chosen over other potential inhibitors of TGFβ-1 and CXCR4? Was this based on existing literature, structural suitability, or availability? A stronger justification would improve clarity.
2. The proposed dual inhibition strategy using D4476 and AMD3100 lacks a mechanistic explanation of how these drugs act synergistically. More details are needed on their combined effect on downstream signalling pathways.
3. The study lacks a direct comparison with currently approved breast cancer therapeutics. How does the dual inhibition strategy perform in comparison to standard treatment modalities? Addressing this would strengthen the clinical relevance of the findings.
4. Given that drug resistance is a major hurdle in breast cancer treatment, the manuscript should discuss potential resistance mechanisms that could arise from targeting TGFβ-1 and CXCR4.
5. The therapeutic strategy involves inhibition of TGFβ-1 and CXCR4, which are implicated in multiple physiological processes. A discussion on potential off-target effects and toxicity risks is essential.
6. The manuscript should clarify whether datasets were independently validated. If publicly available datasets were used, were they cross-checked with multiple cohorts to ensure reproducibility?
7. Some sections contain awkward phrasing and grammatical inconsistencies. A thorough revision for language precision is required to improve readability.
