# Supplementary material for: Unravelling the therapeutic potential of dual TGFβ-1 and CXCR4 inhibition in breast cancer using computational strategies
Source: PLoS One. 2025 Dec 5;20(12):e0323665. doi: 10.1371/journal.pone.0323665 (PMC12680324; doi:10.1371/journal.pone.0323665)
Supplement: Attachment — Submitted filename: Response to Reviewers.docx [file pone.0323665.s002.docx]

**Rebuttal Letter**

**Response to Reviewers:**

Reviewer #1: This study, “Exploring soluble mediators in Breast Cancer: Expression Dynamics of Transforming Growth Factor Beta 1 and C-X-C Chemokine Receptor Type 4 and the Therapeutic Opportunities” (Manuscript ID: PONE-D-25-01591) explores the role of selected soluble mediators—TGFβ-1, CXCR4, IL19, BMP1, VCAN, and WNT2—in breast cancer (BC) through extensive in silico bioinformatics analyses. The authors leverage multiple databases and tools (UALCAN, TIMER 2.0, TISCH2, DISCO, STRING, Enrichr, etc.) to assess gene expression, molecular pathways, and cellular localization across pan-cancer and breast cancer datasets. They then use molecular docking, DFT, and MD simulations to propose a synergistic therapeutic approach targeting TGFβ-1 and CXCR4 using inhibitors D4476 and AMD3100.

While the manuscript is elaborate and data-rich, I believe it requires some revisions before it can be considered for publication. The primary concerns include:
1. The study presents a thorough computational analysis integrating gene expression profiling, pathway enrichment, protein-protein interaction networks, and molecular docking/dynamics simulations. The combination of TGFβ-1 and CXCR4 inhibition using D4476 and AMD3100, respectively, is a potentially valuable therapeutic strategy. However, the study would benefit from a more precise articulation of how this approach significantly advances current knowledge in the field, as much of the data is derived from publicly available resources without novel experimental validation.

**Response:** Dear reviewer thanks you for your suggestions. The succeeding comments have been addressed in detail, which collectively clarify how our study advances current knowledge.

2. The current title is long and contains multiple technical terms that could overwhelm or confuse readers. Simplifying the title and focusing it around the core theme—synergistic inhibition of TGFβ-1 and CXCR4 in breast cancer using in silico approaches—would make it more impactful and accessible.

**Response:** We appreciate your valued feedback and modified the title per your suggestion. The updated version of the manuscript contains the modified title as:**"**Unraveling the Therapeutic Potential of Dual TGFβ-1 and CXCR4 Inhibition in Breast Cancer Using Computational Strategies."
3. The abstract should be revised to improve logical flow and cohesiveness. Currently, it reads like a list of disconnected observations. A well-structured abstract should provide (i) a clear background and rationale, (ii) major methods or analytical tools used, (iii) key findings, and (iv) a concise conclusion emphasizing the significance of the study. Connecting these components will give readers a more complete understanding of the study’s objectives and outcomes.

**Response:** Dear Reviewer, thank you for your insightful feedback. We have revised the abstract to enhance its logical flow and cohesiveness.

4. The methodology section, particularly subsections 2.3 (bc-GenExMiner), 2.5 (GeneMANIA), and 2.8 (UCSC Xena), lacks adequate detail on the specific parameters, datasets, and filtering criteria used. For reproducibility, please describe how data was queried, which cancer subtypes were selected, whether normalization or statistical thresholds were applied, and how results were interpreted. Explicit mention of software versions, database release dates, and settings (e.g., algorithms used in GeneMANIA or TIMER) is strongly recommended.

**Response:** Dear Reviewer, we have modified the subsections of the methodology to include details on data querying, cancer subtypes, normalization methods, statistical thresholds, software versions, and algorithm settings to ensure reproducibility.

5. While the study proposes dual inhibition of TGFβ-1 and CXCR4 as a synergistic therapeutic approach, it does not contextualize this within the landscape of existing standard-of-care therapies. It would strengthen the manuscript to include a comparison with current treatment strategies for breast cancer (e.g., hormone therapy, HER2-targeted therapy, checkpoint inhibitors) to help evaluate the novelty and translational potential of this approach.

**Response:** We thank the reviewer for this excellent suggestion, and we have now added a separate paragraph on this dual inhibition strategy in the Introduction and Discussion sections of the paper to relate it to the current standard-of-care therapies for breast cancer.

6. Although the study identifies interactions between key genes such as TGFβ-1, CXCR4, BMP1, VCAN, and WNT2, their functional relevance in tumor progression, immune evasion, or therapy resistance is not adequately discussed. The authors should expand on the biological significance of these interactions, ideally supported by literature or data-driven insights, to give the findings more mechanistic depth.

**Response:** We thank the reviewer for this thoughtful suggestion. In view of this, we have expanded the Discussion section to consider further the functional relevance of the key genes identified—IL-19, TGFβ-1, CXCR4, BMP1, VCAN, and WNT2—with some consideration given here to their mechanistic roles in the areas of tumor progression, immune evasion, and resistance to therapy.

7. To improve the translational impact of the work, the authors should elaborate more clearly on how their findings could influence breast cancer diagnosis, prognosis, or treatment. For example, could the co-targeting of TGFβ-1 and CXCR4 be tailored to specific breast cancer subtypes (e.g., TNBC or luminal B)? Are the expression levels of these targets associated with patient survival or therapeutic response?

**Response:** The suggestion put forward by the reviewer is much appreciated. In this response, we extended the discussion section to make clear the possible clinical relevance of our findings. In detail, we discuss the co-targeting of TGFβ-1 and CXCR4 as potentially suited for aggressive subtypes such as triple-negative breast cancer (TNBC), with expression levels of these genes serving as prognostic indicators or therapeutic targets.

8. The Results and Discussion sections would benefit greatly from concise summary or concluding remarks at the end of major subsections. This would help readers synthesize the findings and understand their importance before moving to the next topic. It also creates a more cohesive narrative flow throughout the manuscript.

**Response:** We thank the reviewer for this highly appreciated suggestion. We would like to mention that in responding to the previous comments--upon which we focused, we put in place focused summary statements at the close of respective subsections in both Results and Discussion.

9. Several figures (especially Figures 1–9) lack informative legends and clear axis labels. Ensure all figures are self-explanatory, with well-defined titles, labels, and consistent formatting. It would also help to include brief descriptions of what each figure demonstrates in the text body, especially when transitioning between panels.

**Response:** Dear Reviewer, we have revised the legends for Figures to ensure that they contain informative legends and are self-explanatory.

10. The manuscript contains numerous grammatical and syntactic errors, awkward phrasing, and typographical issues that hinder readability. A professional language and editing review is highly recommended.

**Response:** Thank you for pointing this out. The manuscript has now been thoroughly revised for grammar, syntax, and overall readability.

11. Synergistic targeting of TGFβ-1 and CXCR4 is a known concept; this manuscript repeats rather than advances it.

**Response:** Even with the existence of the idea, our research offers a novel perspective on breast cancer in terms of subtype-specific expression, multi-pathway correlations, and their potential role in treatment resistance, which provides previously unrecognised translational relevance.

12. The statistical analysis is unclear (e.g., there are no p-values, no confidence intervals, and no quantitative validation).

**Response:** We appreciate the comment. We have already included statistical measures wherever necessary, including p-values, local clustering coefficients, and other relevant quantitative validations within the figures and results.

13. Most figures do not add much value to the manuscript, so they can be put as supplementary.

**Response:** We are grateful for the recommendation. On the other hand, we think the figures are crucial for clarifying and presenting important results. They aid in more successfully communicating complex facts and provide direct support for the key findings. For this reason, we would rather keep them in the main manuscript.

**Reviewer #2:** Upon reviewing the manuscript entitled "Exploring Soluble Mediators in Breast Cancer: Expression Dynamics of Transforming Growth Factor Beta 1 and C-X-C Chemokine Receptor Type 4 and the Therapeutic Opportunities," I have pinpointed a number of minor improvements that the authors could make-in order to enhance the general quality of the paper. The suggested revisions focus on improving the overall clarity, data interpretation, and presentation of the manuscript, ultimately enhancing its readability and scientific rigor.

1. Why were D4476 and AMD3100 chosen over other potential inhibitors of TGFβ-1 and CXCR4? Was this based on existing literature, structural suitability, or availability? A stronger justification would improve clarity.

**Response:** D4476 and AMD3100 were selected according to pre-existing literature evidence as well as target specificity, structural suitability, and availability for in vitro studies. D4476 is a well-characterized inhibitor that acts indirectly to inhibit TGF-β signalling through inhibition of ALK5-mediated SMAD2 phosphorylation, while AMD3100 is an FDA-approved CXCR4 antagonist that effectively blocks CXCR4-mediated signalling in numerous cancers. Their use in the study is justified due to their well-established pharmacological profiles and suitability with breast cancer models, making them excellent candidates for exploring dual inhibition strategies.

2. The proposed dual inhibition strategy using D4476 and AMD3100 lacks a mechanistic explanation of how these drugs act synergistically. More details are needed on their combined effect on downstream signalling pathways.

**Response:** Thank you for the suggestion. D4476 inhibits TGFβ-1/SMAD2/3 signalling, while AMD3100 blocks the CXCL12/CXCR4 axis. Together, they disrupt EMT, migration, and survival pathways (e.g., PI3K/AKT, MAPK), leading to a synergistic anti-tumor effect. We have now added this mechanistic explanation to the Discussion section.

3. The study lacks a direct comparison with currently approved breast cancer therapeutics. How does the dual inhibition strategy perform in comparison to standard treatment modalities? Addressing this would strengthen the clinical relevance of the findings.

**Response:** We thank the reviewer for this excellent suggestion, and we have now added a separate paragraph on this dual inhibition strategy in the Introduction and Discussion sections of the paper to relate it to the current standard-of-care therapies for breast cancer.

4. Given that drug resistance is a major hurdle in breast cancer treatment, the manuscript should discuss potential resistance mechanisms that could arise from targeting TGFβ-1 and CXCR4.

**Response:** Thank you for this insightful suggestion. We recognize that potential resistance mechanisms to TGFβ-1 and CXCR4 inhibition were not addressed. A short discussion has now been added in the manuscript to elaborate on adoptive responses and compensatory signalling that may limit long-term efficacy, thus emphasizing the necessity for combinations or sequential therapies.

5. The therapeutic strategy involves inhibition of TGFβ-1 and CXCR4, which are implicated in multiple physiological processes. A discussion on potential off-target effects and toxicity risks is essential.

**Response:** Thank you for raising this important point. We have now included a brief discussion on the possible toxicity risks and the need for targeted delivery strategies to minimize adverse effects.

6. The manuscript should clarify whether datasets were independently validated. If publicly available datasets were used, were they cross-checked with multiple cohorts to ensure reproducibility?

**Response:** I appreciate your insightful feedback. We confirm that every dataset utilised in this research was openly accessible. Gene expression and pathway studies were cross-validated across several independent datasets and platforms, such as TIMER2.0, UALCAN, TISCH2, UCSC Xena, and DISCO, to guarantee repeatability and robustness.

7. Some sections contain awkward phrasing and grammatical inconsistencies. A thorough revision for language precision is required to improve readability.

**Response:** Thank you for pointing this out. The manuscript has now been thoroughly revised for grammar, syntax, and overall readability.
