# Supplementary material for: Unravelling the therapeutic potential of dual TGFβ-1 and CXCR4 inhibition in breast cancer using computational strategies
Source: PLoS One. 2025 Dec 5;20(12):e0323665. doi: 10.1371/journal.pone.0323665 (PMC12680324; doi:10.1371/journal.pone.0323665)
Supplement: Attachment — Submitted filename: Response to Reviewers plos.docx [file pone.0323665.s003.docx]

Reviewer #1: The authors have addressed my comments. I still want authors to proofread the manuscript for English and grammatical errors as I could find a lot more even after revision.

**Response:** We appreciate the reviewer’s feedback. The revised manuscript has been thoroughly reviewed and edited by a native-level English language editor to correct all remaining grammatical, syntactic, typographical, and stylistic issues

Reviewer #2: The authors made sufficient changes based on the reviewers comments. Under current modifications this manuscript should be accepted.

**Response:** Thank you for your positive assessment. We appreciate your recommendation and are glad the revisions have met the required standards.

Reviewer #3: This manuscript discusses the issue of breast cancer research, aiming to elucidate the role of key immunomodulatory and signalling genes (TGFβ-1, IL19, CXCR4, BMP1, VCAN, and WNT2) in the disease’s pathogenesis via bioinformatics approaches. The integration of gene expression analysis with therapeutic targeting offers important insights, particularly regarding the synergistic potential of targeting TGFβ-1 and CXCR4. This research required a revision as follows:
1. Please expand the Background on BC and TGF-β, refer this work: <https://doi.org/10.1016/j.saa.2022.122000>, <https://doi.org/10.3788/COL202018.051701>, <https://doi.org/10.1111/cns.14489>, [https://doi.org](https://doi.org/) /10.1245/s10434-024-16454-8

**Response:** We thank the reviewer for this valuable suggestion. We have expanded the Background section in the revised manuscript to provide a more detailed overview of breast cancer and the role of TGF-β signalling, incorporating insights from the suggested references. However, due to technical limitations with the EndNote referencing software, we were unable to insert these references directly through EndNote.

2. Provide clear significance and rationale with research gap of this work.

**Response:** We thank the reviewer for highlighting this important point. In the revised manuscript, we have clearly outlined the significance and rationale of our study by emphasizing the urgent need for novel therapeutic strategies in TNBC, where current treatment options are limited.
3. Expand the methods: Summary of databases/tools used (e.g., GEPIA, TIMER, STRING).

**Response:** We thank the reviewer for this suggestion. We have already addressed this comment in the revised manuscript by providing a comprehensive summary of all databases and tools used, including GEPIA, TIMER, STRING, UALCAN, bc-GenExMiner, TISCH2, Enrichr, DISCO, and UCSC Xena.

4. What platforms were used for expression and survival analysis (e.g., TCGA, GEPIA, UALCAN)?

**Response:** Expression and survival analyses were performed using platforms including UALCAN, TIMER 2.0, bc-GenExMiner, and UCSC Xena, primarily utilizing TCGA datasets.
UALCAN and bc-GenExMiner were used for expression across clinical subtypes, while TIMER and Xena provided pan-cancer and tumor-specific insights.
These tools enabled evaluation of gene expression, immune infiltration, and clinical correlation of TGFβ-1, IL19, CXCR4, BMP1, VCAN, and WNT2 in breast cancer. Survival analysis was not performed using online tools in this study.
Instead, relevant survival data for the genes of interest were gathered from published literature.
These studies provided supporting evidence on the prognostic significance of TGFβ-1, IL19, CXCR4, BMP1, VCAN, and WNT2 in breast cancer.

5. Were protein–protein interaction (PPI) networks constructed (e.g., STRING, Cytoscape)?

**Response:** Yes, protein–protein interaction (PPI) networks were constructed using the STRING database. The interactions among TGFβ-1, IL19, CXCR4, BMP1, VCAN, and WNT2 were analyzed to identify their functional associations. Cytoscape was used; all visualizations and network analyses were performed directly through the cytoscape platform.

6. Describe the criteria for gene selection, analysis parameters, and software versions.

**Response:** The genes TGFβ-1, IL19, CXCR4, BMP1, VCAN, and WNT2 were selected based on their reported involvement in breast cancer progression and the tumor microenvironment, as supported by prior literature. Default parameters were used for analysis across platforms like UALCAN (v2024), TIMER 2.0, bc-GenExMiner v4.5, STRING (v12.0), and Enrichr. Only TCGA-BRCA datasets were selected where applicable, and significance was considered at *p* < 0.05. All tools were accessed online between January and May 2025.

7. Need more clarify how synergism was inferred. Was it based on gene co-expression, network centrality, or drug sensitivity databases?

**Response:** Synergism was not directly inferred from co-expression, network centrality, or drug sensitivity databases. Instead, literature evidence and in silico analyses showed that both drugs individually had promising anticancer activity. Based on these findings, we proceeded to explore their potential synergistic effect in combination, supported by gene and pathway analyses**.**

8. Pan-cancer relevance (any significance in other cancers should be contextualized) in discussion

**Response:** We have addressed this comment in the revised manuscript by incorporating a discussion on the pan-cancer relevance of the selected genes.

9. Provide, Pathway enrichment analyses (e.g., GO, KEGG)

**Response:** Dear reviewer, we have already incorporated the pathway enrichment analyses in the manuscript using Enrichr, including both GO (BP, MF, CC) and KEGG pathway results.
10. How dual inhibition might work mechanistically—any supporting preclinical or clinical data?

**Response:** Based on literature and in silico findings, simultaneous targeting of pathways involving cell proliferation (e.g., TGFβ signaling) and drug resistance/immune modulation (e.g., CXCR4, IL19) can exert complementary effects, leading to enhanced antitumor activity. Supporting preclinical studies have demonstrated that combining agents affecting these pathways can overcome resistance, reduce tumor growth, and improve therapeutic efficacy in various cancers, including breast cancer.
11. The possible role of IL19, BMP1, VCAN, and WNT2—are these primarily pro-tumorigenic, or context-dependent?

**Response:** Based on current literature, IL19, BMP1, VCAN, and WNT2 are primarily considered pro-tumorigenic, promoting processes like inflammation, ECM remodeling, cell migration, and Wnt signaling. However, their roles can be context-dependent, varying with tumor type, microenvironment, and stage,

12. Address limitation, potential false positives or overinterpretation of bioinformatics findings.

**Response:** We have acknowledged the limitations of our study in the revised manuscript, particularly the reliance on publicly available bioinformatics data, which may introduce false positives or overinterpretation.

Reviewer #4: This manuscript presents an integrative computational analysis to explore the potential of dual inhibition of TGFβ-1 and CXCR4 as a therapeutic strategy in breast cancer. The authors use various in silico platforms to assess gene expression profiles, protein-protein interaction networks, pathway enrichment, and molecular docking/dynamics simulations. The study suggests that dual targeting with D4476 and AMD3100 may offer synergistic anti-tumor effects, especially in aggressive subtypes like TNBC.

Major Concerns
1. The idea of dual inhibition of TGFβ-1 and CXCR4 is not novel and has been previously discussed in the literature. Authors must clarify how their computational findings provide novel mechanistic insights, especially regarding subtype-specific or pathway-level differences not reported earlier.

**Response:** We agree that dual inhibition of TGFβ-1 and CXCR4 has been discussed previously; however, the unique combination of genes explored in our study—including IL19, BMP1, VCAN, and WNT2—alongside TGFβ-1 and CXCR4, and the use of a comprehensive in silico pipeline involving functional enrichment, PPI networks, and pan-cancer expression profiling, has not been reported earlier. Our approach provides novel mechanistic insights, especially into subtype-specific roles and interactions relevant to triple-negative breast cancer, offering a fresh perspective for therapeutic targeting.

2. The study remains entirely in silico. While computational analyses are comprehensive, the translational value remains speculative without in vitro or clinical validation. At minimum, authors should discuss plans for experimental follow-up and acknowledge the limitations of drawing therapeutic conclusions solely from in silico work.

**Response:** We acknowledge this limitation and have included a statement in the revised manuscript addressing the purely in silico nature of our study.

3. While the authors propose synergy between D4476 and AMD3100, the mechanistic basis at the pathway level is not rigorously dissected. Add a schematic diagram and a detailed pathway map showing how the inhibition of TGFβ and CXCR4 signalling converge on key EMT, CSC, or metastasis pathways.

**Response:** We thank the reviewer for this valuable suggestion. In the revised manuscript, we have included a schematic diagram and detailed pathway map illustrating how dual inhibition of TGFβ (via D4476) and CXCR4 (via AMD3100) may converge on key cancer-related processes such as EMT, cancer stem cell maintenance, and metastasis.
4. The manuscript lacks robust statistical metrics. P-values, confidence intervals, or validation across cohorts are not consistently reported. Please improve the statistical rigor in gene expression and docking analyses by indicating effect sizes, p-values, and confidence intervals where appropriate.

**Response:** We acknowledge the reviewer’s concern regarding statistical metrics. Since the analyses were performed using established bioinformatics portals (e.g., UALCAN, TIMER, bc-GenExMiner), the results presented are based on pre-processed datasets, and only statistically significant outputs (typically with p-values < 0.05) were extracted and reported. These platforms apply internal statistical tests and thresholds, and we have now clarified this in the revised manuscript.

5. Expand figure legends to include the context, sample sizes, data source, and interpretation of key trends. Use arrows or color-coded annotations to highlight significant findings.

**Response:** We thank the reviewer for this suggestion. We have already addressed this comment while responding to other reviewers by expanding the figure legends.

6. The translational potential (e.g., application to TNBC, resistance cases, or prognosis) is mentioned but not convincingly argued. Clearly define how co-targeting TGFβ-1 and CXCR4 could be integrated into existing treatment paradigms, and whether this is more suitable for specific molecular subtypes.

**Response:** We thank the reviewer for this important point. We have already addressed this comment in the revised manuscript under the Discussion section, while responding to Reviewer 1 and Reviewer 2. Specifically, we elaborated on the translational potential of co-targeting TGFβ-1 and CXCR4, particularly in TNBC and therapy-resistant cases, and discussed how this strategy could be integrated into current treatment paradigms based on molecular subtype-specific vulnerabilities.

7. The rationale for choosing D4476 and AMD3100 is weak, and potential off-target effects are not adequately addressed. Please provide literature-based justification for drug selection and discuss anticipated toxicological risks in normal tissues expressing TGFβ-1 and CXCR4.

**Response:** We thank the reviewer for this observation. We have already discussed the rationale for selecting D4476 and AMD3100, along with the potential off-target effects and toxicological risks, in the revised manuscript under the Discussion section, while addressing related comments from Reviewers 1 and 2

Minor Issues:
1. A native-level language editor should review English grammar, syntax, and phrasing. Numerous typographical and stylistic issues remain.

**Response:** We appreciate the reviewer’s feedback. The revised manuscript has been thoroughly reviewed and edited by a native-level English language editor to correct all remaining grammatical, syntactic, typographical, and stylistic issues.

2. Use gene/protein nomenclature consistently (e.g., “CXCR4” vs “Cxcr4”).

**Response:** We thank the reviewer for pointing this out. In the revised manuscript, we have ensured consistent use of gene and protein nomenclature throughout, following standard conventions (e.g., human genes in uppercase italics, such as *CXCR4*, and proteins in uppercase regular font, such as CXCR4). All gene and protein names have been reviewed and corrected accordingly.
